# Epithelial and mesenchymal stem cell biology in the mammary gland: mechanisms, comparative insights, and translational perspectives

**DOI:** 10.3389/fcell.2026.1982804

**Published:** 2026-10-23

**Authors:** Itamar Barash

**Affiliations:** Institute of Animal Science, ARO, The Volcani Center, Rishon LeZiyyon, Israel

**Keywords:** epithelial stem cells, mammary gland, mesenchymal stem cells, mTOR, stem cell niche

## Abstract

Mammary stem cell biology provides an important link between tissue regeneration, mammary development, metabolic adaptation, inflammation, and lactational function. This review examines epithelial and mesenchymal stem cell populations in the mammary gland, with emphasis on their complementary roles and interactions within the tissue microenvironment. Evidence from murine, human, and bovine studies is integrated to highlight common biological principles as well as species-specific features relevant to mammary function and disease. Particular attention is given to the dynamic interactions among epithelial, stromal, immune, endocrine, and metabolic components of the mammary niche and to how these interactions regulate stem cell activity and tissue homeostasis. Metabolic regulation is illustrated by the role of mechanistic target of rapamycin complex 1 (mTORC1) in mammary epithelial stem cell regulation. Evidence from the mammary gland indicates that intermittent mTORC1 inhibition can increase stem-cell activity, with potential effects on mammary development, lactational capacity, and persistency, although effects on whole-animal milk production remain to be established. Mesenchymal stem cells are considered both as resident mammary stromal populations and as potential external mediators of epithelial–stromal communication. They may integrate metabolic, endocrine, and inflammatory signals and contribute to tissue homeostasis and repair. Their therapeutic potential is further discussed in the context of bovine mastitis, where mesenchymal stem cell-based approaches remain experimental and may act through paracrine, immunomodulatory, and experimentally supported antimicrobial mechanisms. Collectively, current evidence supports distinct but complementary roles of epithelial and mesenchymal stem-cell populations in mammary development, regeneration, and tissue maintenance, although their identity and functional significance remain incompletely resolved across species. Defining these populations and their interactions may inform stem cell-based approaches to mammary health and regeneration in humans and to health and production in farm animals.

## Graphical Abstract

**Figure:**
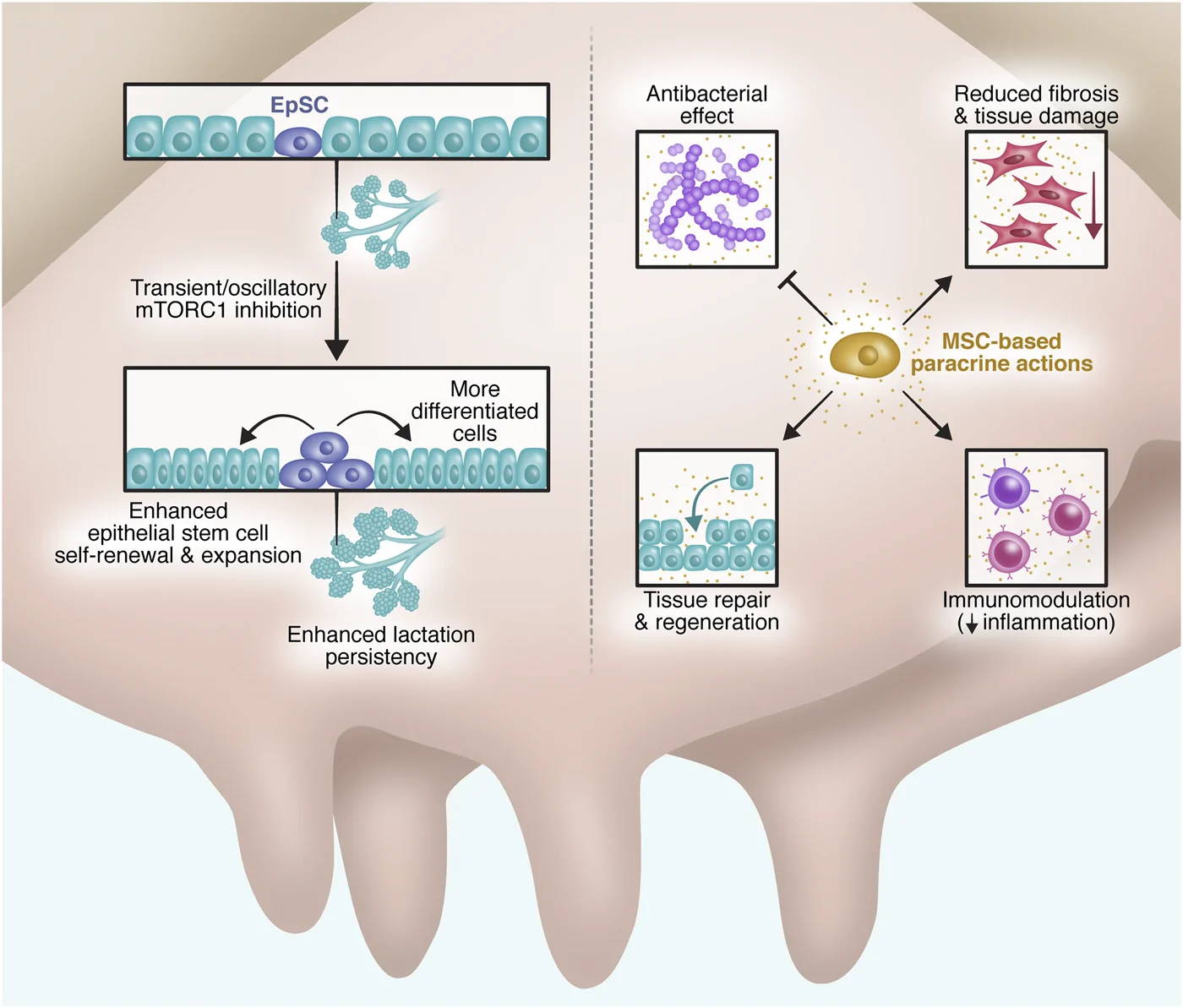
Integrated regulation of mammary stem-cell function and mammary health. This graphical abstract summarizes key insights emerging from the comparative analysis of mammary stem-cell biology in murine, human, and bovine systems. The bovine mammary gland provides a physiologically relevant large-animal context for examining how epithelial stem cells (EpSCs) and mesenchymal stem cells (MSCs) contribute complementary roles to mammary tissue function and health**.** Transient or oscillatory mTORC1 inhibition is supported by experimental evidence as a means of enhancing EpSC self-renewal and expansion, potentially supporting subsequent epithelial differentiation and lactational persistency. In parallel, experimental studies indicate that MSCs act through paracrine and immunomodulatory mechanisms to promote tissue repair and regeneration, limit fibrosis and tissue damage, and contribute to antibacterial and anti-inflammatory responses, supporting their potential therapeutic applications**.** Together, these complementary activities illustrate how regulation of the epithelial stem-cell compartment and the mesenchymal microenvironment can shape mammary function, regeneration, and health, providing a foundation for translational strategies to improve mammary health in humans and health and production in bovines.

## 1 Introduction: mammary gland biology and stem-cell populations

The mammary gland is a specialized exocrine organ that reallocates maternal resources for milk production. Its function is tightly integrated with systemic physiology and is highly responsive to endocrine, metabolic, and immune signals. Epithelial cells execute milk production and secretion, whereas mesenchymal and immune components shape the local microenvironment through structural support, immunomodulation, and paracrine signaling. Milk production depends on dynamic cycles of mammary remodeling, driven by coordinated epithelial turnover and regeneration.

Since the domestication of dairy species approximately 8,000–9,000 years ago, milk production has become a cornerstone of agriculture and human nutrition, driving co-evolutionary adaptations such as lactase persistence in human populations (Curry, 2013; Evershed et al., 2008). However, rising demand for milk is still met largely through herd expansion rather than improved biological efficiency (OECD/FAO, 2021), highlighting the need for mechanistically informed approaches to mammary function. At the cellular level, these processes are ultimately rooted in stem-cell biology**.**

Positioned at the apex of cellular hierarchies, stem cells are defined by their ability to self-renew and differentiate into specialized cell types (Lajtha, 1979). During mammary development, embryonic precursor cells give rise to tissue-specific epithelial and mesenchymal stem cells that acquire properties suited to the mammary tissue (Slack, 2008). Spatial and temporal cues during development establish the adult tissue-specific niches where stem cells persist.

The pivotal role of epithelial stem cells (EpSCs) in regenerating the mammary parenchyma between lactation cycles and in maintaining both normal and pathological tissue homeostasis has been extensively investigated. Broadly, these cells give rise to basal and luminal lineages: the basal lineage generates contractile myoepithelial cells, which surround the epithelial cells that form the ductal and alveolar compartments.

The parenchymal epithelium is embedded within a stroma, the fibrotic-to-fatty composition of which is species specific and affects metabolic and structural functions. This stromal compartment contains resident mesenchymal stem cells (MSCs), which differentiate into supportive cell types that exert regulatory and immunomodulatory effects (Pittenger et al., 2019). In contrast to mammary EpSCs, the biological properties and potential applications of their mammary MSC counterparts, whether resident or externally introduced**,** remain comparatively underexplored. This distinction is particularly relevant because MSCs may represent both resident mammary stromal populations and externally introduced cells used experimentally or therapeutically. Here, the evidence for epithelial and mesenchymal stem-cell populations in the mammary gland is examined, with emphasis on their biological properties, interactions with the tissue environment, species-specific features, and potential relevance to mammary regeneration, health, and production.

## 2 Mammary EpSC biology: conceptual lessons from murine models

The mammary gland exhibits a unique postnatal developmental program characterized by extensive remodeling across successive cycles of pregnancy, lactation, and involution. This regenerative capacity is supported by a rare mammary EpSC populations together with more abundant lineage biased progenitors that contribute both basal/myoepithelial and luminal lineages. Functional identification of these cells relies primarily on transplantation assays, in which mammary epithelial cells are introduced into an epithelium-depleted (“cleared”) mouse fat pad (Deome et al., 1959). Landmark transplantation-based studies demonstrated that single or small numbers of cells enriched for basal markers (e.g., CD49f^high^CD24^med^ or CD29^high^CD24^+^) can regenerate a fully functional gland (Stingl et al., 2006; Shackleton et al., 2006), providing the experimental foundation for identifying mammary epithelial populations with stem-cell activity.This approach has been refined using additional markers, including H2b (dos Santos et al., 2013), CD200 and its receptor (Rauner et al., 2018), Axin2 (van Amerongen et al., 2012), Procr (Wang et al., 2015), LGR5 (Fu et al., 2017), Dll1 (Chakrabarti et al., 2018) as well as indicators of WNT and Notch signaling activity (Zhou et al., 2019; Bouras et al., 2008). Collectively, these markers support the presence of phenotypically and functionally distinct EpSC populations. Complementary *in vitro* assays, such as mammosphere formation under non-adherent conditions, capture stem cell–associated properties (Dontu et al., 2003; Dontu et al., 2004).

During early morphogenesis, the outer cap-cell layer of the terminal end bud (TEB) contains approximately 260 EpSCs, including at least 35 stem/progenitor cells that contribute to ductal morphogenesis (Scheele et al., 2017; Davis et al., 2016). Following puberty, as terminal end buds regress, the anatomical localization of EpSCs becomes less well defined, with evidence supporting their dispersed distribution along the ductal network, where they remain poised to respond to hormonal and local cues.

A central unresolved issue concerns the extent to which adult EpSC populations retain multilineage potential under physiological conditions**.** Early transplantation-based studies supported a model of bipotent stem cells capable of generating both basal and luminal lineages. However, lineage-tracing approaches in intact mammary tissue challenged this view, showing that in the adult gland, basal and luminal compartments are largely maintained by distinct, lineage-restricted stem/progenitor populations (Van Keymeulen et al., 2011; Wuidart et al., 2016). Adult maintenance is therefore proposed to follow largely lineage-restricted trajectories toward basal/myoepithelial or luminal lineages (Scheele et al., 2017; Davis et al., 2016). These divergent conclusions reflect differences in experimental context. Transplantation assays, which involve dissociation and ectopic engraftment, may reveal latent plasticity not expressed within the native niche. Conversely, lineage-tracing approaches preserve tissue context but depend on lineage-specific promoter (e.g., CK14, CK18) activity and proliferative status, potentially underrepresenting rare or quiescent populations (Rios et al., 2016). Subsequent advances combining lineage tracing with three-dimensional imaging have suggested the possible persistence of rare bipotent cells; however, their functional contribution to normal tissue maintenance remains uncertain (Rios et al., 2014). More recently, integrated single-cell transcriptional, spatial, and lineage-tracing analyses have further refined this reconciliation model by revealing marked heterogeneity within the mammary basal compartment. CD34^−^CD200^+^ basal cells, enriched near the nipple region, exhibited the strongest long-term regenerative activity, whereas CD34^+^CD200^−^ cells associated with TEBs displayed more restricted progenitor behavior linked to alveologenesis. Lineage tracing identified Bcl11b^+^ cells within the CD34^−^CD200^+^ population as rare long-lived bipotent stem cells contributing to both basal and luminal lineages during postnatal homeostasis and across repeated pregnancies, while Sema3a^+^ cells behaved predominantly as rapidly responding unipotent progenitors. Collectively, these findings support a model in which rare bipotent stem cells coexist with more abundant lineage-biased progenitors, although the quantitative contribution of bipotent cells to normal adult mammary homeostasis remains unresolved (Lin et al., 2025).

Despite the controversies, several principles are well established. EpSCs are largely devoid of classical hormone receptors and respond indirectly to systemic signals through paracrine interactions with neighboring epithelial and stromal cells. Key pathways include Wnt, Notch, EGFR, IGF, and RANK signaling (Fu et al., 2020). In parallel, the stem cell niche, comprising extracellular matrix components, immune cells, and mesenchymal populations, plays a central role in governing EpSC fate (Rauner and Kuperwasser, 2021). To this end, progress in the field remains constrained by the absence of a definitive mammary stem cell marker, analogous to LGR5 in the intestine (Barker et al., 2007) and by the lack of comparable lineage-tracing approaches outside experimental murine models. Although markers such as CD200, Bcl11b, and Sema3a provide increasing resolution of mammary epithelial heterogeneity, none uniquely defines all regenerative populations across developmental states. A comparative summary of the experimental approaches used to identify and characterize mammary stem-cell populations across species, together with their strengths and limitations, is provided in Table 1.

**TABLE 1:** Comparative overview of mammary epithelial stem cell biology in mouse, bovine, and human mammary glands.

Feature	Mouse	Human	Bovine
Functional evidences for EpSC activity	Strong evidence from fat-pad transplantation/reconstitution, serial transplantation, clonogenic assays, and lineage tracing (Stingl et al., 2006; Fu et al., 2017; Dontu et al., 2003; Lin et al., 2025)	Clonogenic, organoid, and xenotransplantation studies identify populations with repopulating and/or bipotent potential (Stingl et al., 2001; Eirew et al., 2008; Linnemann et al., 2015)	Regenerative and morphogenic activity demonstrated by transplantation/xenotransplantation of EpSC-enriched populations. Functional heterogeneity is supported (Rauner and Barash, 2012; Rauner and Barash, 2016; Kosenko et al., 2022a)
Lineage resolution	Lineage tracing and reporter approaches have defined basal, luminal, and progenitor relationships (Van Keymeulen et al., 2011; Rios et al., 2016; Lin et al., 2025)	Single-cell and molecular profiling has refined epithelial heterogeneity, but functional and lineage relationships remain less directly resolved than in mouse (Regan and Smalley, 2020; Gray et al., 2023)	Limited. Hierarchical relationships are inferred from transplantation, marker-defined populations, label retention, clonogenicity, and transcriptional analyses; direct *in vivo* lineage tracing is unavailable (Capuco, 2007; Rauner and Barash, 2012; Rauner and Barash, 2016; Rauner et al., 2013)
Identification of EpSC enriched populations	CD24, CD29, CD49f, CD61, and Sca-1, together with lineage reporters and many study-specific markers described in the text (Stingl et al., 2006; Shackleton et al., 2006; Fu et al., 2020; Vaillant et al., 2008; Welm et al., 2002)	EpCAM and CD49f distinguish basal, luminal-progenitor, and mature luminal populations. ALDH, MUC1, keratins, single-cell, and organoid approaches provide additional characterization (Stingl et al., 2001; Eirew et al., 2012)	CD24 and CD49f are used for enrichment; ALDH is associated with early progenitor/stem-like properties. CD24medCD49fpos cells have been proposed as EpSC-enriched (Martignani et al., 2010; Rauner and Barash, 2012)
Interpretation of marker expression	Markers can enrich for functionally defined populations, but expression alone does not establish stem-cell function; transplantation and lineage analyses provide critical validation (Stingl et al., 2006; Shackleton et al., 2006; Lin et al., 2025)	Markers reveal epithelial heterogeneity and progenitor states but should not automatically be equated with functional stem cells; studies differ regarding population location and potency (Visvader and Stingl, 2014)	Marker-defined populations aid enrichment and characterization, but their hierarchical and regenerative status is less firmly established than in mouse (Rauner and Barash, 2012; Rauner and Barash, 2016)
Stromal/Niche context	Robust epithelial regeneration and analysis of epithelial–stromal interactions (Stingl et al., 2006; Shackleton et al., 2006; Zhou et al., 2019) No reciprocal interspecies transplantation studies	Epithelial organization is affected by tissue and stromal context. Human epithelium show limited colonization in murine mammary fat pad unless human stromal environment is provided. Organoids provide a complementary but incomplete model of the native environment (Kuperwasser et al., 2004; Proia and Kuperwasser, 2006; Rosenbluth et al., 2020)	Strong stromal dependence. Bovine epithelium shows incomplete morphogenesis in the murine fat pad, whereas bovine stroma can improve engraftment and duct-like organization (Rauner and Kuperwasser, 2021; Kosenko et al., 2022a)
Scientific value in this review	Principal mechanistic and lineage-resolved reference for mammary epithelial stem/progenitor organization	Important translational reference for human mammary health and disease	Principal large-animal and translational context, relevant to mammary productivity, lactation, and mammary health
Major knowledge gaps	Quantitative contributions of defined stem/progenitor populations during homeostasis, pregnancy, aging, and tumorigenesis remain incompletely resolved (Visvader and Stingl, 2014; Lee et al., 2026)	Relationship between molecularly defined populations, long-term regenerative activity, and lineage behavior in the intact breast remains incompletely resolved (Visvader and Stingl, 2014)	Direct *in vivo* lineage tracing is lacking; definitive stem cells remain difficult to distinguish from progenitor-enriched populations, and their contribution to lactational function remains incompletely established (Capuco, 2007; Rauner and Barash, 2012; Rauner and Barash, 2016)
Key comparative question	Which mechanisms of epithelial maintenance and lineage regulation are conserved across species?	Which mechanisms identified in experimental models are conserved in the human breast and relevant to health and disease?	Which features of epithelial hierarchy and niche dependence are species-specific versus conserved, and how do they relate to mammary production?
Current research orientation	Mechanistic analysis of epithelial hierarchy, lineage regulation, signaling pathways and stem/progenitor function	Increasing use of single-cell, spatial, organoid, and molecular approaches to define epithelial and stromal heterogeneity and its relevance to health and disease	Increasing emphasis on epithelial–stromal interactions, metabolic and physiological regulation, productivity, and mammary health

References in the table are representative; additional references supporting the statements are provided in the main text.

Together, mammosphere formation, clonogenicity, marker expression, label retention, and transplantation provide complementary evidence of stem-cell properties, while additional *in vivo* functional and lineage-resolved approaches enable a more comprehensive assessment of stem-cell identity and activity. The integration of these complementary approaches is particularly important for defining the lineage potential of mammary EpSC populations and their contribution to tissue maintenance. Important questions remain regarding their functional hierarchy and lineage plasticity, as well as their relationship to disease susceptibility, production, and broader translational relevance.

## 3 Human mammary epithelial stem/progenitor cells: from functional studies to single-cell analysis and breast cancer

Studies of human mammary epithelial stem/progenitor cells have relied largely on marker-based isolation, together with functional assays such as clonogenicity, organoid formation, and xenotransplantation. These approaches provide evidence for regenerative and lineage potential but do not directly establish stem-cell identity in the intact human breast. Unlike in the mouse, genetic lineage tracing cannot be performed in the human mammary gland, leaving the contribution and lineage relationships of putative stem/progenitor populations *in situ* less directly resolved (Visvader and Stingl, 2014; Stingl et al., 2001). Transplantation studies have nevertheless demonstrated that prospectively isolated human epithelial populations can display substantial regenerative and multilineage potential, although the transplantation process itself alters the cellular microenvironment and therefore assesses this potential under experimentally perturbed conditions (Stingl et al., 2001; Eirew et al., 2008) The importance of the microenvironment to stem/progenitor cell behavior is illustrated by humanization studies showing that human mammary epithelial cells poorly colonize the murine mammary fat pad unless an appropriate human stromal environment is provided, emphasizing the contribution of epithelial–stromal interactions to mammary epithelial organization and function (Kuperwasser et al., 2004; Proia and Kuperwasser, 2006). Thus, marker expression and regenerative capacity in surrogate assays cannot necessarily be interpreted as evidence for a discrete, autonomous stem-cell population or its activity in the native human breast. These limitations, together with the considerable heterogeneity of the human epithelium, have provided an important rationale for applying single-cell and spatial approaches to define epithelial cell states and their relationships. Table 1 summarizes the principal experimental evidence, lineage-resolution approaches, marker-based strategies, and stromal/niche considerations that distinguish human mammary stem/progenitor studies from the more lineage-resolved mouse literature.

Recent single-cell studies have substantially refined our understanding of human mammary epithelial stem/progenitor populations by revealing greater diversity of epithelial cell states than could be resolved using conventional surface markers alone. Early single-cell RNA-sequencing analysis of normal human breast tissue identified distinct basal and luminal epithelial populations and suggested transcriptional trajectories linking basal and luminal states, including populations with features of luminal progenitors (Nguyen et al., 2018). Subsequent transcriptional profiling of human breast stem/progenitor-enriched populations demonstrated considerable heterogeneity within ALDH-positive and CD44^+^CD24^−^ populations, emphasizing that commonly used markers do not define homogeneous stem-cell compartments (Colacino et al., 2018).

Importantly, single-cell analyses have revealed a more complex and continuous spectrum of epithelial cell states than implied by a simple hierarchical model, with overlapping and transitional transcriptional states along epithelial differentiation trajectories (Nguyen et al., 2018; Regan and Smalley, 2020; Wahl and Spike, 2017). These findings support a more fluid concept of epithelial cell states and plasticity.

More recent large-scale single-cell and spatial transcriptomic analyses have greatly expanded this picture, identifying numerous epithelial states and placing putative progenitor populations within their anatomical and stromal context. For example, a spatially resolved atlas of the adult human breast profiled 714,331 cells from 126 women and identified 58 biological cell states, revealing extensive heterogeneity among luminal epithelial populations (Kumar et al., 2023). These studies support the view that human mammary epithelial stem/progenitor biology is characterized by substantial cellular heterogeneity and transcriptional plasticity rather than by sharply separated populations defined by individual markers.

Importantly, single-cell analyses have increasingly been complemented by functional approaches, although such evidence remains relatively limited. Human mammary organoid studies have shown that basal/stem-enriched and luminal progenitor populations can generate mature epithelial lineages in culture (Rosenbluth et al., 2020). A multimodal single-cell breast atlas further demonstrated that subtypes of the major epithelial lineages can be maintained in organoid cultures (Gray et al., 2023). Together, these studies illustrate the complementary value of molecular and functional approaches, with the latter providing evidence of regenerative and lineage-generating capacity. More refined xenotransplantation approaches that better incorporate the human epithelial–stromal context may further strengthen the functional assessment of candidate populations.

Interestingly, single-cell analyses of human milk have provided a non-invasive view of mammary epithelial populations during lactation. These studies identified predominantly luminal epithelial cells, including populations with transcriptional features resembling luminal progenitors (Martin Carli et al., 2020; Twigger et al., 2022). However, such transcriptional profiles do not establish *bona fide* stem-cell properties, and functional evidence for self-renewal and multilineage regenerative capacity of human milk-derived cells remains limited.

Functional studies of breast cancer have provided important evidence for tumor-initiating cell populations. A landmark study by Al-Hajj and colleagues demonstrated that as few as 100 Lin^−^ CD44^+^CD24^-^/^low^ cells isolated from human breast tumors were capable of initiating tumors following transplantation into immunodeficient mice, whereas much larger numbers of cells with alternative phenotypes were not (Al-Hajj et al., 2003). These findings led to the hypothesis that breast cancers may arise from mammary stem cells or early progenitor cells that acquire oncogenic mutations (Zhou et al., 2019; Morrison et al., 2008; Ercan et al., 2011). However, the precise cellular origin of these cancers remains controversial, as the proposed cells share many molecular and functional characteristics with normal mammary stem cells. Moreover, accumulating evidence indicates that the cell of origin differs among breast cancer subtypes; for example, BRCA1-associated basal-like tumors arise from luminal epithelial progenitors rather than basal stem cells (Molyneux et al., 2010).

Taken together, human studies have extended the experimental framework established in murine mammary EpSC research through functional assays, transplantation, organoid models, and increasingly sophisticated single-cell and spatial approaches, while revealing substantial epithelial heterogeneity and dependence on tissue context. However, the precise contribution of defined epithelial stem/progenitor populations to normal tissue maintenance and carcinogenesis, as well as their potential for therapeutic manipulation, remains incompletely resolved.

## 4 Bovine mammary EpSC biology: organizational principles, microenvironmental constraints and context-dependent readouts compared with murine systems

Bovines constitute the primary source of milk for human consumption and have been extensively characterized with respect to mammary gland morphology and function through more than a century of lactation research (Akers, 2017). Before key murine studies using basal–luminal reporter systems provided functional evidence for mammary stem cells (Stingl et al., 2006; Shackleton et al., 2006), progenitor-like cells had already been proposed to contribute to the shape and persistence of the bovine lactation curve (Capuco et al., 2003). However, in contrast to the murine system, where a coherent epithelial hierarchy has been established through functional assays and lineage tracing, progress in defining the cellular organization and regulation of bovine mammary EpSCs and progenitor cells has remained comparatively limited.

Because lineage tracing in the bovine gland is not currently available, a bovine mammary EpSC is best regarded operationally as an epithelial population with evidence of sustained regenerative and/or multilineage capacity, supported by complementary functional and phenotypic approaches rather than by definitive *in situ* lineage-resolved criteria (Table 1).

Early observations provided conceptual support for hierarchical organization of the epithelial compartment in the bovine mammary gland. Studies identified a population of small, pale-staining epithelial cells with high proliferative activity, initially interpreted as candidate stem or progenitor cells based on their cytological features and spatial distribution (Ellis et al., 2002; Chepko and Smith, 1997). In retrospect, this population is now understood to largely represent actively cycling progenitors rather than a stem cell pool. Additional support for a putative stem-like compartment emerged from label-retaining assays: BrdU pulse–chase experiments identified a small fraction of slowly cycling epithelial cells (0%–5%) with low proliferative activity and heterogeneous hormone receptor expression that were unevenly distributed across the external, mid, and cisternal regions of the bovine mammary gland (Capuco, 2007). While consistent with candidate stem cell features (Potten et al., 1978), these observations remain inferential and do not establish hierarchical position or regenerative capacity, highlighting a key distinction from murine studies in which stem cell identity is defined functionally *in vivo*.

More recent work has attempted to resolve epithelial heterogeneity using marker-based approaches. Cells with high ALDH activity display properties associated with early progenitor or stem-like states, including enhanced clonogenicity (Martignani et al., 2010). In parallel, fractionation based on marker-assisted sorting using CD24 and CD49f, which are selectively expressed in the luminal and basal layers, respectively, on Lin-sorted cells, proved suitable for isolating bovine EpSC-enriched populations (Rauner and Barash, 2012). These analyses support a hierarchy in which CD24^med^CD49f^pos^ cells occupy an apex and give rise to basal (CD24^neg^CD49f^pos^) and luminal (CD24^high^CD49f^neg^) progenitors that subsequently differentiate into mature CD24^med^CD49f^neg^ luminal epithelial cells. Functional heterogeneity within these compartments is further supported by colony-forming assays and transcriptional profiling (Rauner and Barash, 2012; Rauner and Barash, 2016), which distinguish early and late progenitor states—broadly paralleling the murine epithelial hierarchy, albeit with less definitive functional validation. Accordingly, CD24^med^CD49f^pos^ cells are more appropriately described as an EpSC-enriched population, rather than as definitively established stem cells.

A recurring and defining feature of bovine EpSC enriched populations is its strong dependence on the microenvironment. Xenotransplantation experiments demonstrate that EpSC-enriched bovine populations can generate bilayered epithelial structures with key features of differentiation, including lumen formation, cytokeratin patterning, and hormone receptor expression in the de-epithelialized (cleared) mouse mammary fat pad. However, these structures typically remain spherical and fail to undergo ductal elongation within the murine mammary fat pad, indicating an incomplete morphogenetic response in a heterologous niche (Rauner and Barash, 2016; Rauner et al., 2013). Attempts to modify the stromal environment, including co-transplantation with fibroblasts, increase engraftment rates, but do not restore ductal architecture. Similarly, murine–bovine chimeric approaches have largely failed to produce integrated outgrowths, although a single instance of terminal duct lobular unit (TDLU)-like formation under estrogen and progesterone stimulation suggested limited structural plasticity (Rauner et al., 2013). These findings indicate that the regenerative and morphogenetic capacity of bovine epithelial populations is strongly affected by the host microenvironment and cannot be fully expressed or assessed in a heterologous murine niche.

Consistent with a requirement for species-specific signals, transplantation into pre-established bovine stromal environments partially rescues this phenotype, resulting in improved engraftment and more elongated, duct-like structures, although full architectural and functional recapitulation was not achieved. This suggests an instructive role for the niche in directing bovine epithelial organization. Complementary *in vitro* studies further highlighted the critical effect of the microenvironment: adipose-conditioned medium markedly inhibited bovine mammosphere expansion, whereas FGF supplementation restored growth, emphasizing the antagonistic balance between inhibitory adipose signals and supportive fibrotic stromal signals (Rauner et al., 2013).

Notably, within bovine stroma, a thin “close stroma” layer at the parenchyma–stroma interface most effectively supports stem cell engraftment and ductal outgrowth, a feature not described in the mature murine gland. This spatially restricted compartment exhibits molecular and functional properties consistent with a specialized niche including features associated with stromal re-epithelialization, and may represent a preferential site at which bovine EpSCs interact with their supporting microenvironment (Kosenko et al., 2022a).

As in the murine field, a central unresolved question concerns the differentiation potential of adult bovine enriched EpSCs. Transplantation-based assays indicate that CD24^med^CD49f^pos^ EpSC-enriched cells can generate both basal and luminal lineages. Stat5 was identified as a later luminal progenitor regulator, and E-cadherin and ZEB1 as markers associated with luminal maturation (Rauner and Barash, 2016). However, this approach does not resolve whether individual stem cells are bipotent or whether multilineage output reflects a mixture of lineage-restricted progenitors. Evidence for basal-restricted outgrowths supports the existence of unipotent populations (Rauner and Barash, 2016), yet their contribution to normal tissue maintenance remains unclear. This uncertainty contrasts with the murine system, where lineage-tracing studies in intact tissue have demonstrated with bipotency demonstrated during embryonic development and also reported in rare long-lived adult populations (Van Keymeulen et al., 2011; Lin et al., 2025). In bovines, the absence of comparable *in situ* lineage-resolution approaches leaves the balance between multipotent and unipotent models unresolved (Table 1). A recent single-cell and single-nucleus transcriptomic analysis has further resolved the cellular heterogeneity of the bovine mammary gland, identifying distinct luminal secretory, hormone-responsive luminal, and basal/myoepithelial populations. However, this analysis does not by itself establish stem-cell identity or regenerative capacity, which requires complementary functional evidence (Han B. et al., 2025). These data therefore provide important resolution of bovine epithelial cell states, but not lineage-resolved evidence for a bovine stem-cell hierarchy.

The dynamics of the EpSC population across physiological states remain similarly incompletely defined. In mouse models, increases in stem cell abundance during pregnancy (Asselin-Labat et al., 2010), no change (Britt et al., 2009) and decrease (Siwko et al., 2008) have all been reported. Equivalent data in cattle remain limited, and changes in bovine mammary EpSCs enriched populations throughout the parity cycle, including puberty, pregnancy, lactation, involution and dry-off are largely unexplored. One study reported that CD49f^high^CD24^pos^ enriched EpSCs, comprised approximately 3.3% of total mammary cells during puberty and declined progressively by ∼50% between puberty and lactation, ultimately reaching 0.4% during the dry period (Finot et al., 2019). Another study demonstrated a decrease in bipotent luminal cells during lactation (Perruchot et al., 2016). Thus, by comparison, murine models provide a more detailed, though still debated view of stage-specific regulation of the stem cell population.

Milk provides a unique, non-invasive interface with the mammary epithelium and has therefore been explored as a potential source for studying epithelial and stem/progenitor cell populations *in vivo*. Milk contains a heterogeneous population of cells arising from multiple physiological processes within the gland, including epithelial shedding from alveolar and ductal compartments, apoptotic cell loss during late lactation and involution and active trafficking of immune cells such as macrophages, neutrophils, and lymphocytes (Webster et al., 2024; Lemay et al., 2013; Li et al., 2014). Transient disruptions in epithelial integrity and paracellular transport may further facilitate cellular entry into the lumen. Together, these mechanisms generate a complex cellular milieu containing epithelial and immune cells in varying physiological states, including viable, apoptotic, senescent, and other transitional or degenerative states. Within this context, stem cell–like populations have been identified in milk across species. Human milk was shown to contain embryonic-like cells with multilineage differentiation potential (Hassiotou et al., 2012). Subsequent marker-based analyses identified bovine milk–derived populations with mixed stem–progenitor characteristics alongside differentiated epithelial and myoepithelial cells (Pipino et al., 2018; Danev et al., 2023). Colostrum contains epithelial, mesenchymal, and hematopoietic stem-like populations, but their abundance declines rapidly after parturition; epithelial stem-like cells, for example, decrease from ∼0.9% to ∼0.1% within the first 12 h (Finot et al., 2025). Interpretation of these findings remains limited, however, because detachment from the native mammary microenvironment may alter cellular state and differentiation potential. Consequently, *ex vivo* properties may reflect displacement-induced plasticity rather than authentic *in situ* stem cell behavior. Furthermore, the heterogeneous mechanisms governing cellular release into milk complicate the assignment of origin, lineage identity, and functional relevance. External factors such as milking frequency may further influence milk cellular composition (Boutinaud et al., 2015). Thus, while milk-derived cells offer an appealing and accessible system for probing mammary epithelial biology, current evidence remains insufficient to define their physiological role or to reliably infer stem cell function within the intact gland. Rather, these observations reinforce a central theme of bovine EpSC biology: cellular properties are highly context-dependent, critically shaped by the microenvironment, and not readily extrapolated outside the native niche. In this respect, the interpretational limitations associated with milk-derived populations reflect broader challenges in the field, where experimental accessibility is often achieved at the expense of physiological context.

Taken together, current evidence supports the existence of a hierarchical and functionally heterogeneous epithelial system in the bovine mammary gland, comprising candidate and enriched stem-cell populations with evidence of regenerative and multilineage capacity. However, relative to the murine model, this system remains less precisely defined, strongly constrained by its microenvironment, and more difficult to interrogate *in vivo* (Table 1). These differences highlight a fundamental trade-off between experimental tractability and physiological relevance: murine models provide mechanistic resolution and conceptual basis, whereas bovine systems capture the regulatory complexity of a highly productive organ operating under sustained metabolic demand. Defining bovine EpSCs more precisely will require integration of phenotypic, functional, and spatial approaches that can distinguish true stem-cell properties from those of lineage-biased progenitors.

Bridging this gap will be essential for advancing both fundamental understanding and application.

### 4.1 Experimental strategies to modulate self-renewal and expansion of bovine mammary EpSC-enriched populations

In oncology, mammary EpSCs are considered important therapeutic targets because they may survive conventional therapies, thereby contributing to tumor recurrence and disease relapse (Yang et al., 2017; Chang, 2016; Son et al., 2024; Lindeman and Visvader, 2010). By contrast, bovine mammary EpSC research is directed toward expanding this cell population to support epithelial turnover, maintain mammary tissue function, and ultimately enhance milk production. This rationale is grounded in the low proliferative index of the bovine mammary epithelium during the extended lactation and the progressive loss of differentiated secretory cells that parallels declining yield (Capuco et al., 2001). Accordingly, strategies that promote stem cell self-renewal, particularly when performed during the unmilking “dry period” during late gestation, may enhance lactational persistency (Figure 1).

**FIGURE 1:**
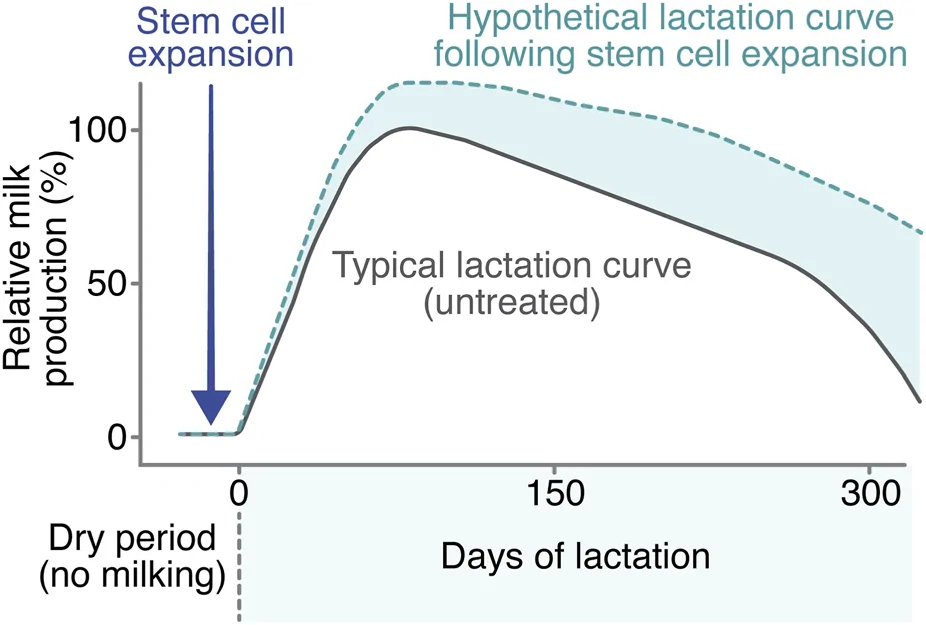
Proposed conceptual model linking mammary epithelial stem cell (EpSC) augmentation during late gestation—a period corresponding to the unmilked dry period in dairy cows—to potentially enhanced lactation performance. The solid black curve represents a typical lactation profile of an average Holstein cow, whereas the dashed blue curve illustrates a hypothetical lactation profile following expansion of the mammary EpSC pool during the dry period. Increased EpSC abundance is proposed to support subsequent progenitor differentiation and the generation of additional secretory epithelial cells during lactation, potentially contributing to increased lactation persistency. The marked area between the curves represents the conceptual potential for increased cumulative milk production associated with enhanced maintenance of the epithelial compartment. The curves and the shaded area are conceptual and do not represent measured lactation data or a quantitatively predicted increase in milk yield or lactation persistency.

In 3-month-old calves, a developmental stage predictive of adult mammary function (Kosenko et al., 2022b), intramammary xanthosine delivery increased label-retaining epithelial cells and telomerase activity (Capuco et al., 2009). However, these indirect measures were not accompanied by functional evidence of expansion of the mammary stem cell pool. Downstream translational production outcomes in goats were negligible (Choudhary et al., 2018), and subsequent studies failed to demonstrate consistent effects on clonogenicity, canonical stem/progenitor markers, or regenerative capacity. Instead, delayed global suppression of proliferation was observed (Rauner and Barash, 2014), consistent with possible perturbation of nucleotide homeostasis (Kozhevnikova et al., 2012). Thus, despite a plausible biochemical entry point, the currently available evidence does not yet provide strong support for mechanistic specificity at the stem cell level or for robust translational efficacy in the mammary gland.

More recent approaches have shifted toward metabolic control of stem cell fate, focusing on the mechanistic target of rapamycin (mTOR) pathway as an integrator of nutrient status and cell fate decisions. At the molecular level, mTOR integrates upstream inputs from cellular energy status through AMP-activated protein kinase (AMPK), amino acid availability and growth factor signaling (PI3K–AKT) (Inoki et al., 2012; Sancak et al., 2008; Manning and Toker, 2017). AMPK functions as an energy sensor that generally restrains mTORC1 activity when cellular energy is limited, thereby linking metabolic state to anabolic growth and cell fate.

mTOR operates through two functionally distinct complexes: mTOR complex 1 (mTORC1), which regulates protein synthesis via S6K1 and 4E-BP1, ribosome biogenesis, and anabolic metabolism; and mTOR complex 2 (mTORC2), which modulates AKT signaling, cytoskeletal organization, and survival (Zoncu et al., 2011).

Calorie restriction (CR) provides an important example of how systemic metabolic status can regulate stem-cell activity through the mammary niche. By reducing mTORC1 activity in niche cells, CR can indirectly promote stem-cell self-renewal. In the intestine, this mechanism involves reduced mTORC1 signaling in stem-cell-adjacent Paneth cells and BST-1-dependent production of the paracrine mediator cyclic adenosine diphosphate ribose (cADPR) (Yilmaz et al., 2012). A comparable response has been observed in the mammary gland: in both the mouse mammary gland and bovine mammary xenografts implanted into the de-epithelialized mammary fat pad of immunocompromised mice, CR increased stem-like activity, as reflected in mice by a higher take rate of transplanted mammary stem cells and greater occupancy of the fat pad by epithelial outgrowths, and in bovine mammary cultures by increased mammosphere formation, while suppressing differentiation markers (Kfir and Barash, 2019). These findings are consistent with a shift toward self-renewal and indicate that the response is not exclusively epithelial-intrinsic. Notably, the mammary response also appears to involve niche-mediated BST-1 production (Kfir and Barash, 2019), supporting the concept that stromal and immune components can function as metabolic sensors that translate systemic nutritional cues into signals regulating EpSC behavior (Kosenko et al., 2022c).

Translationally, these observations provided a rationale for pharmacological approaches aimed at reproducing selected CR-induced effects on stem-cell activity without imposing the systemic metabolic burden of dietary restriction. However, the mode and temporal pattern of mTOR inhibition proved critical. Continuous rapamycin exposure, which enforces sustained mTORC1 suppression, reduces global protein synthesis and can impair tissue function (Jankiewicz et al., 2006). In contrast, transient or oscillatory inhibition, as discussed below, may preserve translational capacity while periodically relieving mTORC1-driven differentiation pressure. Although the downstream events have not been directly examined in mammary EpSCs, transient mTORC1 inhibition could induce reversible dephosphorylation of S6K1 and 4E-BP1, temporarily reducing ribosome biogenesis and cap-dependent translation. Reactivation of mTORC1 would then restore phosphorylation of these targets and translational activity. This proposed mechanism provides a plausible explanation for how intermittent modulation might influence the balance between self-renewal and differentiation without imposing sustained suppression of protein synthesis, but it remains a mechanistic model rather than an established process in bovine mammary EpSC-enriched populations (Igarashi and Guarente, 2016; Igarashi and Guarente, 2017).

In line with this model, intermittent rapamycin administration in mouse mammary models induced oscillatory mTORC1 inhibition, increased the stem cell fraction (∼1.5-fold), and promoted ductal outgrowth, while maintaining protein synthesis. Related effects were observed in bovine mammary implants, where enhanced epithelial regeneration and increased milk-protein gene expression were observed (Kfir and Barash, 2019), (Figure 2A).

**FIGURE 2:**
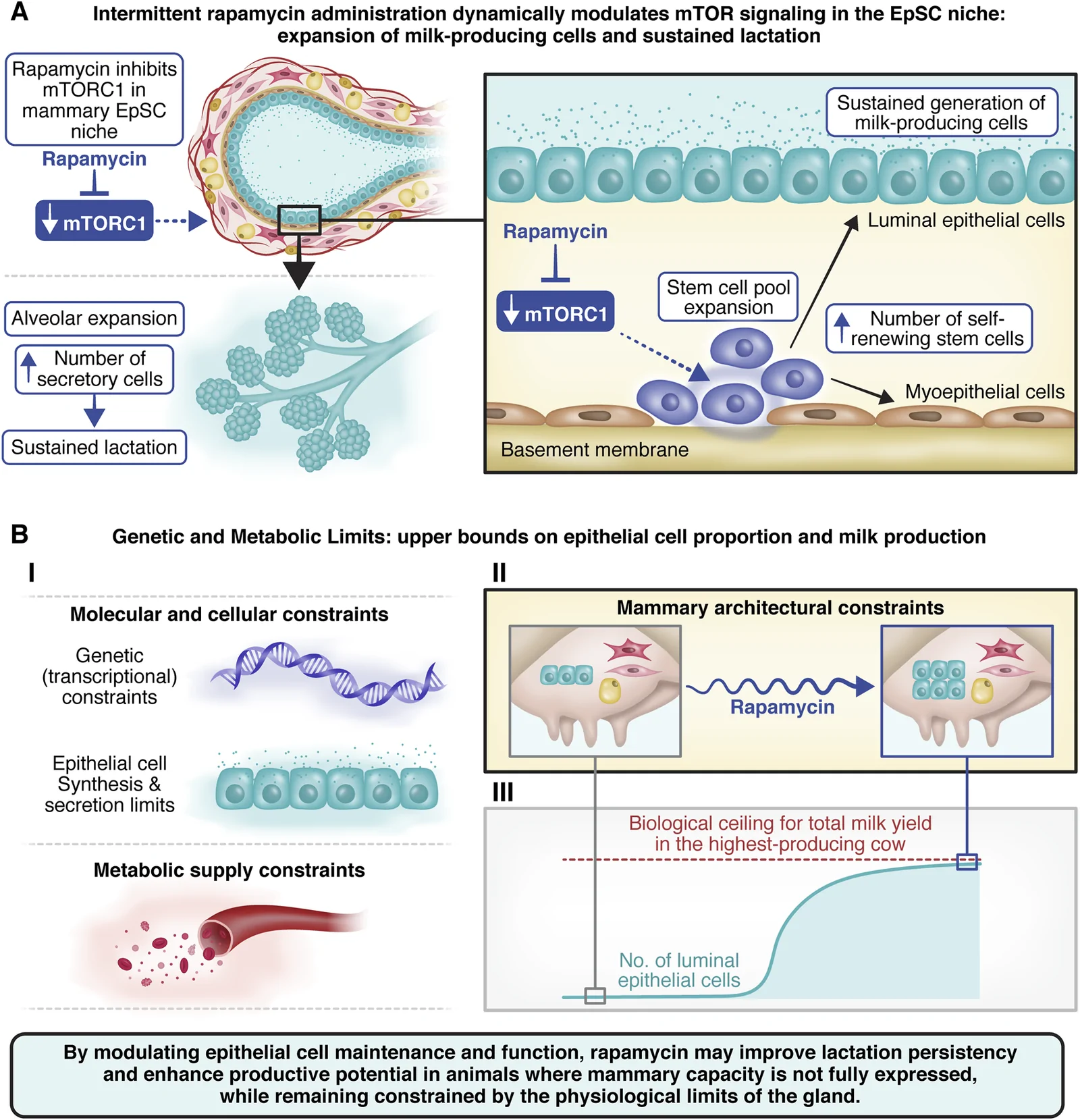
Conceptual model of rapamycin-mediated mTORC1 oscillation effect and the hypothetical biological ceiling of mammary milk production. **(A)** Conceptual model for the effects of rapamycin-mediated oscillation of mechanistic target of rapamycin complex 1 (mTORC1) activity within the mammary epithelial stem cell (EpSC) niche. Oscillatory mTORC1 activity was associated with expansion of the mammary stem-cell pool in the mouse. An enlarged stem-cell pool, followed by proliferation and differentiation was followed by mammary tissue development in the experimental systems examined. In the mouse, this sequence was accompanied by alveolar expansion and the generation of additional milk-producing epithelial cells, whereas bovine systems showed comparable epithelial responses that extended further to the induction of milk-protein gene expression. These findings provide a basis for the hypothesis that stem-cell expansion and subsequent differentiation could contribute to maintenance of the secretory epithelium and sustained milk-protein production during lactation. Importantly, this proposed effect is attributed primarily to increased numbers of milk-producing epithelial cells rather than increased milk protein gene expression per individual cell. **(B)** Conceptual model illustrating the integrated biological constraints that may impose an upper limit on milk production. These include: (I) intrinsic genetic and transcriptional constraints, encompassing the maximal transcriptional output of milk protein genes and their regulatory accessibility; epithelial biosynthetic and secretory capacity, encompassing protein synthesis, folding, processing, intracellular trafficking, and secretion; and metabolic supply limitations, encompassing amino acid and energy availability, nutrient partitioning, and mammary blood flow. (II) We further propose that architectural and physiological constraints of the mammary gland may limit the expansion of the luminal mammary epithelium following stem-cell augmentation and downstream progenitor production, thereby constraining expansion of the epithelial compartment within the mammary stroma and, consequently, milk production. (III) Together, these factors are proposed to define a hypothetical biological ceiling for milk production in the mammary gland. The model and the proposed ceiling are conceptual and do not represent measured or quantitatively fitted limits of milk production.

Building on these mechanistic insights, intramammary delivery in calves tested transient mTORC1 modulation in a large-animal context. An intermittent “skip-a-day” regimen, designed to approximate oscillatory mTOR dynamics, achieved partial (∼50%) mTORC1 inhibition without compromising tissue architecture or milk-protein expression (Kosenko et al., 2022b). This transient inhibition elicited a delayed but durable increase in stem-cell self-renewal activity, reflected by a ∼2.6-fold increase in epithelial cell proliferation *in vitro* (Kosenko et al., 2022b). It was accompanied by a 31% increase in epithelial cell proliferation *in vivo* and up to sixfold induction of milk-protein gene expression *in vivo*, based on comprehensive analysis of mammary tissue collected from the treated animals (Kosenko et al., 2022b). The delayed kinetics are consistent with a model in which transient shifts in stem-cell division symmetry gradually propagate through the epithelial hierarchy to modulate tissue-level functional output, although this interpretation has not been directly demonstrated experimentally (Kosenko et al., 2022b). Notably, the increase in milk-protein gene expression is proposed to reflect an expansion of the secretory epithelial compartment relative to the mesenchymal compartment, rather than enhanced milk-protein expression on a per-cell basis, and therefore represents increased milk-synthetic capacity at the tissue level rather than a demonstrated increase in whole-animal milk yield, which remains the ultimate production outcome to be established in future studies (Figure 2A). Consistent with the existence of intrinsic limits on epithelial cell output (Figure 2B,I), transgenic mice overexpressing the β-lactoglobulin gene failed to achieve proportional increases in total milk-protein synthesis despite robust transgene overexpression, which increased β-lactoglobulin production at the expense of other milk proteins (Wilde et al., 1992).

Importantly, the magnitude of the rapamycin effect was inversely correlated with each calf’s intrinsic milk-protein production potential and occurred for both whey and casein proteins, suggesting that mammary glands converge toward a comparable physiological ceiling of milk-synthetic capacity, probably due to limitations on alveoli expansion, with larger responses in lower-producing glands than in those already operating near this limit (Figure 2B,II,III). This observation further suggests that the potential production benefit of EpSC modulation may depend on the pre-existing functional state of the gland rather than being uniform across animals. Despite these advances, key mechanistic gaps remain. Rapamycin did not fully recapitulate CR-induced signaling, including induction of BST-1/cADPR pathways, suggesting that mTOR-independent or parallel metabolic circuits contribute to the full CR phenotype (Kfir and Barash, 2019). Moreover, emerging data implicate macrophages as critical niche sensors, with CSF1-mediated signaling and polarization toward an M2-like state linking energy status to EpSC regulation (Kosenko et al., 2022c). These findings reinforce the concept that effective translational strategies must account for multicellular metabolic integration rather than epithelial-intrinsic pathways alone. Overall, intermittent local modulation of mTORC1 represents a promising experimental strategy for expanding mammary epithelial regenerative capacity and potentially improving the production potential of the bovine mammary gland. The substantial stem-cell and milk-protein responses observed in calves provide a rationale for further development of this approach as a potential means of improving cattle production. However, it remains an experimental strategy: its effects on actual milk yield, lactational persistency, animal health, and long-term safety must be established in appropriately designed studies in lactating cattle before practical application can be considered.

### 4.2 Constraints on translating rapamycin-based mTOR modulation to improved milk production in cattle

The experimental findings described above provide a rationale for transient modulation of mTORC1 during the un-milked dry period preceding parturition as a means of modulating bovine mammary epithelial renewal, but several biological and practical constraints must be addressed before this approach can be considered for improving milk production. Nutritional regulation of mTOR is one potential route, as mTOR signaling is highly responsive to nutrient availability (Nobukuni et al., 2005) and particularly sensitive to branched-chain amino acids in mouse and bovine mammary epithelial cells (Moshel et al., 2006; Burgos et al., 2010). Conversely, certain amino acid combinations, including lysine, histidine, and threonine, can suppress mTOR activity (Prizant and Barash, 2008). However, nutritional interventions are difficult to control precisely under production conditions and are therefore unlikely to provide the temporal and spatial control required to reproduce the experimental mTORC1 response.

A more direct approach is pharmacological modulation of mTOR. Beyond its immunosuppressive actions, mTOR inhibition modulates autophagy, metabolism, cell growth, and longevity across multiple experimental systems (Ganesh and Devi, 2023; Roark and Iffland, 2025; Liu et al., 2019). In the mammary gland, these pleiotropic effects intersect with stem cell–dependent epithelial turnover, making mTOR an attractive but complex target for intervention. Nevertheless, pharmacological targeting of mTOR presents substantial biological, regulatory, and practical challenges. Sustained mTOR inhibition suppresses protein synthesis and epithelial function, whereas transient or temporally restricted modulation may be required to promote stem cell self-renewal without impairing differentiation. The optimal timing, duration, and magnitude of such modulation remain undefined. The therapeutic window between sufficient pathway modulation to enhance EpSC activity and excessive inhibition that compromises epithelial function remains to be established.

Additional challenges arise from the pharmacological properties of rapamycin itself. Although widely used clinically and approved by the U.S. FDA in 1999 for the prevention of renal transplant rejection, rapamycin is a macrolide immunosuppressant with a prolonged elimination half-life (∼69 h) (Wallgren et al., 2025). Its prolonged systemic persistence and immunosuppressive activity raise important concerns for use in food-producing animals. In dairy cattle, potential application would therefore require assessment of systemic exposure and establishment of maximum residue limits (MRLs) for milk and edible tissues, together with validated withdrawal periods. This process would necessitate comprehensive residue-depletion studies conducted under worst-case exposure scenarios in accordance with veterinary medicinal-product regulations. Importantly, even localized intramammary administration would not eliminate these requirements, as systemic absorption cannot be excluded.

Although regulatory familiarity with rapamycin is increasing, including approved or investigated veterinary applications in pigs, dogs, fish, and more recently cats (Mayorga et al., 2021; Ryave and Coleman, 2025; Dai et al., 2014) and (https://www.fda.gov/animal-veterinary/cvm-updates/fda-conditionally-approves-drug-management-ventricular-hypertrophy-cats), these applications do not address mammary-specific delivery or residue profiles relevant to dairy cattle. Alternative delivery platforms, such as biodegradable nanoparticles or slow-release hydrogels, may reduce systemic exposure but currently offer limited control over dosing dynamics and do not eliminate concerns regarding drug residues. Consequently, practical implementation of intramammary rapamycin administration in dairy cattle would require substantial advances in both delivery technologies and regulatory approval pathways. Taken together, biological constraints and regulatory barriers currently limit the immediate application of EpSC-targeted interventions for enhancing milk production. Nevertheless, the demonstrated effects of intermittent mTORC1 modulation on bovine mammary EpSC activity, epithelial proliferation, and milk-protein gene expression provide a basis for further investigation. The key next steps are to determine whether these tissue-level responses translate into increased milk yield or lactational persistency in lactating cattle, and to optimize timing, dose, and delivery while evaluating systemic exposure, animal health, residues, and long-term effects. Thus, rapamycin-based mTORC1 modulation remains a promising but experimental approach to improving cattle production, rather than an established production intervention.

## 5 Mesenchymal stem cells: origin, functional properties, mammary stromal specialization and broad agricultural applications

MSCs were first identified by Friedenstein et al. (Friedenstein et al., 1970) as stromal cells within the bone marrow microenvironment that support hematopoiesis (Pittenger et al., 2019). MSCs are a heterogeneous population of plastic-adherent, spindle-shaped cells that, are mesoderm-derived and developmentally and functionally distinct from hematopoietic cells, despite their close anatomical association within the bone marrow niche. Following their initial identification in marrow, MSCs were subsequently isolated from adipose tissue, umbilical cord, placenta, skin, and dental pulp (Pittenger et al., 2019). In addition, MSC-like populations have been derived from human embryonic stem cells and induced pluripotent stem cells, aiming to overcome limitations of adult MSCs such as cellular aging and batch-to-batch variability in translational applications (Tan et al., 2022).

MSCs are classically defined by their immunophenotype, typically expressing CD73, CD90, and CD105, while lacking hematopoietic markers such as CD45, CD34, CD14, and HLA-DR (Lechanteur et al., 2016). In the context of the mammary gland, it is important to distinguish endogenous mesenchymal populations that reside within the tissue from exogenous MSCs that are isolated, expanded, and subsequently administered experimentally. Thus, marker expression and *in-vitro* differentiation capacity provide useful criteria for characterizing MSC populations but do not by themselves establish equivalent stem-cell properties *in vivo*.

MSCs exhibit multilineage differentiation potential, including adipogenic, myogenic, and fibrogenic lineages (Miao et al., 2016; Du et al., 2015), These lineage decisions involve coordinated signaling through TGF-β/BMP, Wnt/β-catenin, MAPK, PI3K/Akt, and Notch pathways (Cheng et al., 2019; Wu et al., 2016; Day et al., 2005). Additional differentiation capacities have also been reported under specific experimental conditions (Komori, 2006; Nakashima et al., 2002; Rosen and Spiegelman, 2001; Tang and Lane, 2012; Yang et al., 2022; Malladi et al., 2007; Yuan et al., 2023; Weintraub et al., 1991; Hernández et al., 2020). However, differentiation observed under experimental conditions does not necessarily establish equivalent differentiation potential or stem-cell behavior *in vivo*. Beyond their differentiation potential, MSCs exert potent immunomodulatory effects that promote resolution of inflammation and tissue repair. They inhibit T- and B-cell proliferation and function, suppress dendritic cell maturation, and modulate innate immune responses, including macrophage polarization toward an anti-inflammatory M2 phenotype (Pittenger et al., 2019; Tan et al., 2022; Lechanteur et al., 2016). NF-κB signaling has been shown to inhibit MSC differentiation and simultaneously promote migration and proliferation in response to pro-inflammatory cues (Kaltschmidt et al., 2021). Collectively, these properties, together with relatively low HLA class I expression reported for MSCs under some conditions, may contribute to relatively low immunogenicity and have supported investigation of allogeneic MSC use in regenerative medicine and immune-mediated diseases, including graft-versus-host disease (GVHD), Crohn’s disease (CD), rheumatoid arthritis (RA) (Lechanteur et al., 2016) and endometrial pathologies (Peng et al., 2026). In addition, MSCs exhibit antimicrobial activity mediated in part by PGE2 and the cathelicidin LL-37 pathway (Krasnodembskaya et al., 2010; Nemeth et al., 2009), further expanding their functional relevance in host defense and tissue repair. These immunomodulatory and trophic properties have contributed to the investigation of MSCs in regenerative and immune-mediated therapies.

In the mammary gland, MSC-related functions can be considered in two distinct contexts. Endogenous mesenchymal populations comprise resident stromal cells and their progeny that form part of the mammary epithelial niche and contribute to tissue organization and homeostasis. In contrast, exogenous MSCs are isolated and expanded from mammary or extra-mammary tissues and subsequently administered experimentally, where they may exert regenerative, immunomodulatory, or trophic effects. These populations should not be considered interchangeable, because resident stromal populations reflect the physiological organization of the mammary gland, whereas the properties and effects of exogenous MSCs are affected by their source, culture conditions, and mode of administration.

### 5.1 Endogenous mesenchymal stem cell populations in the mammary gland

In the mammary gland, mesenchymal stem-cell activity should be considered within a locally maintained stromal compartment composed of resident mesenchymal progenitor/stem-like populations and their differentiated progeny. These cells give rise to fibroblasts, adipocyte and myogenic precursors, perivascular cells, and other extracellular matrix (ECM)-producing populations that collectively establish the structural and signaling architecture of the EpSC niche (Hens and Wysolmerski, 2005; Watson and Khaled, 2008). Although these resident populations share phenotypic and functional features with bone marrow or umbilical cord MSCs, their developmental relationships and stem-cell properties in the intact mammary gland are not necessarily equivalent to those of culture-expanded MSCs. Bone marrow- or prenatally derived MSCs can home to the mammary gland during injury, inflammation, or tumorigenesis (Karnoub et al., 2007; Mishra et al., 2008), but their contribution to the intact gland appears limited (Spees et al., 2016). Thus, the niche-regulatory functions of the mammary stroma are primarily associated with resident stromal populations and their differentiated progeny rather than with recruited MSCs. Within this hierarchy, differentiated stromal populations shape the local microenvironment through paracrine signaling, ECM remodeling, mechanosensing, and responsiveness to hormonal and metabolic cues. Through these activities, they regulate the availability of growth factors, cytokines, and matrix-associated signals that influence EpSC maintenance, proliferation, and differentiation (Paszek et al., 2005; Levental et al., 2009; Weaver et al., 2002; Sumbal et al., 2021; Merlo et al., 2022).

Collectively, stromal regulation provides an important complement to the epithelial stem-cell hierarchy described above. The effects of calorie restriction on mammary stem-cell activity through stromal CSF-1 signaling provide evidence for this regulatory interaction, while suggesting that stromal signals can affect epithelial stem-cell behavior (Kosenko et al., 2022c). Mammary epithelial regeneration therefore depends not only on the intrinsic properties of EpSCs but also on their interactions with a specialized stromal microenvironment. Notably, although human and bovine mammary glands share a lobulo-alveolar architecture, species-specific differences in stromal organization may affect the properties of these resident mesenchymal populations and their responses to endocrine and metabolic signals (Watson and Khaled, 2008; Karnoub et al., 2007; Mishra et al., 2008).

In practice, despite its rich stromal compartment, the bovine mammary gland is rarely exploited as a source of progenitor/stem cells, likely because its resident mesenchymal progenitor populations are specialized niche-resident cells involved in mammary tissue homeostasis rather than a broadly multipotent population readily amenable to isolation and expansion.

### 5.2 Exogenous MSCs: experimental and agricultural applications

Exogenous MSCs have been investigated in animal production primarily for their regenerative, immunomodulatory, and trophic properties. Their applications extend beyond the mammary gland to tissue repair and the treatment of inflammatory or metabolic disorders, although the mechanisms responsible for these effects may involve paracrine and immunomodulatory activity rather than direct tissue replacement.

In animal production, MSC applications extend beyond the mammary gland and are increasingly explored to improve animal health, productivity, developmental programming, and cellular agriculture. They have also been proposed as therapeutic agents to promote recovery from bone and joint injuries and to mitigate metabolic disorders such as ketosis, potentially through transdifferentiation into insulin-producing islet-like cells and modulation of glucose homeostasis (Hill et al., 2019). In parallel, MSCs represent a key cellular platform for cultured meat production. Cultured meat production relies on MSC-derived progenitors to generate the primary tissue components of conventional meat, including muscle, fat, and connective tissue. Accordingly, MSCs have been isolated and characterized from porcine (Kim et al., 2023), ovine (Shah et al., 2023) and bovine (Okamura et al., 2018) sources, with established protocols for *in vitro* differentiation and immunophenotyping. However, large-scale translation remains limited by unresolved safety concerns, relatively reduced myogenic efficiency compared to adipogenic differentiation, challenges in multi-tissue integration, high costs of culture media, and intrinsic constraints in cell-cycle control and differentiation efficiency (Hauser et al., 2024; Reiss et al., 2021; Drela et al., 2019).

In the mammary gland, exogenous MSCs derived from bone marrow, adipose tissue, or other sources have been investigated as experimental interventions for tissue repair, inflammation, and epithelial regeneration. Although bone marrow- or fetal-tissue-derived MSCs can home to the mammary gland during injury, inflammation, or tumorigenesis, the effects of administered MSCs should be distinguished from the physiological functions of endogenous mammary stromal populations. Their therapeutic activity is thought to be mediated largely by paracrine signaling, immunomodulation, extracellular-vesicle-mediated effects, or interactions with the resident stromal and epithelial compartments rather than stable engraftment or differentiation into mammary epithelial cells.

Taken together, the largely paracrine activity of exogenous MSCs provides a mechanistic basis for both their regenerative potential and their context-dependent effects on epithelial, stromal, and immune compartments. Unlike resident mammary stem and stromal populations, these cells are introduced after *ex vivo* expansion and are therefore used primarily to modify the tissue environment rather than to replace endogenous cell populations. This distinction is particularly relevant to their therapeutic use in humans and bovines, where the same trophic, angiogenic, immunomodulatory, and tissue-remodeling properties that support regeneration may also modulate the behavior of the surrounding tissue. The following sections therefore considers the applications of exogenous MSCs in human breast tissue and the bovine mammary gland including the biological and safety considerations associated with their use. The applications of exogenous MSCs in human breast tissue and the bovine mammary gland, together with the biological and safety considerations associated with their use, are discussed in the following sections.

### 5.3 MSC–epithelial crosstalk in the mammary gland: mechanistic basis, oncogenic risk, and relevance to bovine mammary biology

In the mammary gland, EpSCs and mesenchymal stem/progenitor cell populations reside in spatially distinct yet functionally coupled niches that define their functional output and lineage potential (Figure 3). EpSCs are localized within the basal/myoepithelial compartment, where cues from the basement membrane and neighboring epithelial, mesenchymal, and hematopoietic cells regulate self-renewal and differentiation. EpSCs are largely insulated from direct systemic inputs and instead depend on niche-mediated integration of hormonal and metabolic signals. In contrast, mesenchymal stem/progenitor populations are primarily associated with stromal and perivascular compartments (Thottapillil et al., 2025; Yianni and Sharpe, 2019). Recent mammary-gland studies have further implicated vascular-wall mesenchymal stem cells in mammary epithelial–vascular interactions (La Mantia et al., 2025). However, the precise localization and organization of endogenous mesenchymal stem/progenitor populations within the mammary gland remain to be fully established. Within the stromal and perivascular compartments, extracellular matrix composition, mechanical forces, and paracrine mediators regulate their functions in tissue remodeling and immunomodulation (Phinney and Pittenger, 2017; Shabbir et al., 2015). Under homeostatic conditions, they primarily support and regulate the epithelial stem-cell niche. Nevertheless, recent evidence indicates that a PDGFRα^+^ subset of mouse mammary mesenchymal progenitor cells can undergo mesenchymal-to-epithelial transition and contribute *de novo* to both luminal and basal epithelial lineages during mammary morphogenesis, highlighting a context-dependent plasticity within the mammary stromal compartment (Joshi et al., 2019). A critical distinction is between endogenous mammary mesenchymal stem/progenitor populations, studied within their native microenvironment, and exogenous MSCs**,** which are isolated from other tissues and expanded *ex vivo* before administration. These populations are not biologically interchangeable. Following administration, these cells exhibit limited long-term engraftment (Kean et al., 2013) and can act through paracrine signaling and extracellular vesicles rather than durable tissue integration (Han X. et al., 2025).

**FIGURE 3:**
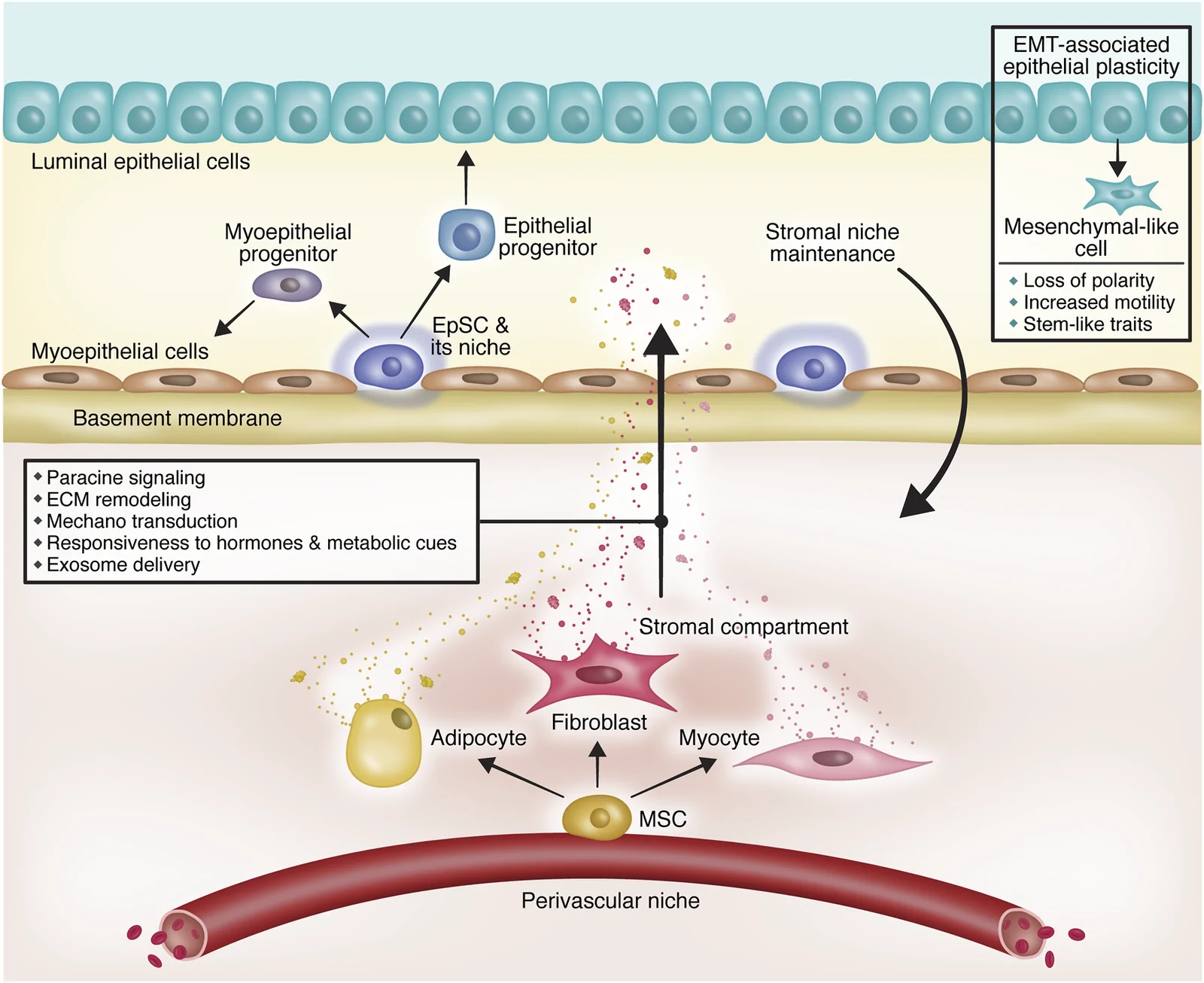
Mesenchymal–epithelial crosstalk in the mammary gland: proposed roles of mesenchymal stem cells. The schematic depicts mammary mesenchymal stem cells (MSCs) within a vascular/perivascular compartment and their potential interactions with epithelial and other stromal cell populations. The depicted MSC–stromal associations are supported by experimental evidence, although their precise organization within the mammary gland is not fully established. MSCs are shown with multilineage differentiation potential, including adipogenic, myogenic, and fibrogenic lineages, and may contribute to mammary tissue homeostasis and epithelial–stromal interactions through the activities depicted in the schematic. The myogenic stromal lineage depicted here is distinct from the epithelial/myoepithelial lineage of the mammary gland. Epithelial to mesenchymal transition (EMT) is illustrated as a context-dependent alteration in epithelial plasticity driven by changes in the stromal microenvironment, including activation of stromal cells, ECM remodeling and stiffening, and disruption of epithelial–stromal interactions. Reciprocal EpSC–MSC interactions and interactions with other stromal populations maintain tissue homeostasis and organization, whereas their dysregulation may contribute to aberrant remodeling and tumor-associated microenvironmental changes. In this schematic, "MSCs” is used in its broad functional sense, encompassing classical MSCs and MSC-like stromal populations described across clinical, veterinary, and experimental systems.

MSC–epithelial communication is mediated through a complex signaling network that includes growth factors (e.g., TGF-β, HGF), cytokines (e.g., IL-6), chemokines (e.g., CCL5), and extracellular matrix remodeling enzymes (Karnoub et al., 2007; Han X. et al., 2025; Zhao et al., 2012). In addition, MSC-derived extracellular vesicles (EVs), have emerged as key effectors capable of transferring miRNAs, proteins, and lipids that influence epithelial proliferation, angiogenesis, lipogenesis and inflammatory responses (Phinney and Pittenger, 2017; Kalluri and LeBleu, 2020; Tkach and Théry, 2016). Co-culture of MSCs with mammary epithelial cells has also been reported to induce epithelial lipogenesis (Tadmor-Levi et al., 2025).

A central process linking MSC activity to epithelial plasticity is epithelial-to-mesenchymal transition (EMT), a reversible program encompassing epithelial, hybrid, and mesenchymal states ((Kalluri and Weinberg, 2009; Thiery et al., 2009) and Figure 4). However, the relationship between EMT and stemness remains highly context dependent. In mammary systems, induction of EMT regulators such as TWIST or SNAIL can confer stem-like properties, including mammosphere formation (Mani et al., 2008), whereas in other contexts mesenchymal traits may not enhance, and may even restrict self-renewal potential (Celia-Terrassa et al., 2012; Sarrio et al., 2012). MSCs can induce EMT in epithelial and carcinoma cells via soluble factors, most prominently TGF-β1, as well as through microenvironmental modulation and direct cell–cell interactions, engaging regulatory axes such as ZEB/miR-200 (Korpal and Kang, 2008; Xu et al., 2012; Takigawa et al., 2017). In breast cancer, EMT-associated signatures have been associated with stem-like traits, therapy resistance, and metastatic relapse, and circulating tumor cells can co-express EMT and stem-cell markers (Klopp et al., 2010; Barriere et al., 2012; Papadaki et al., 2019; Giordano et al., 2012).

**FIGURE 4:**
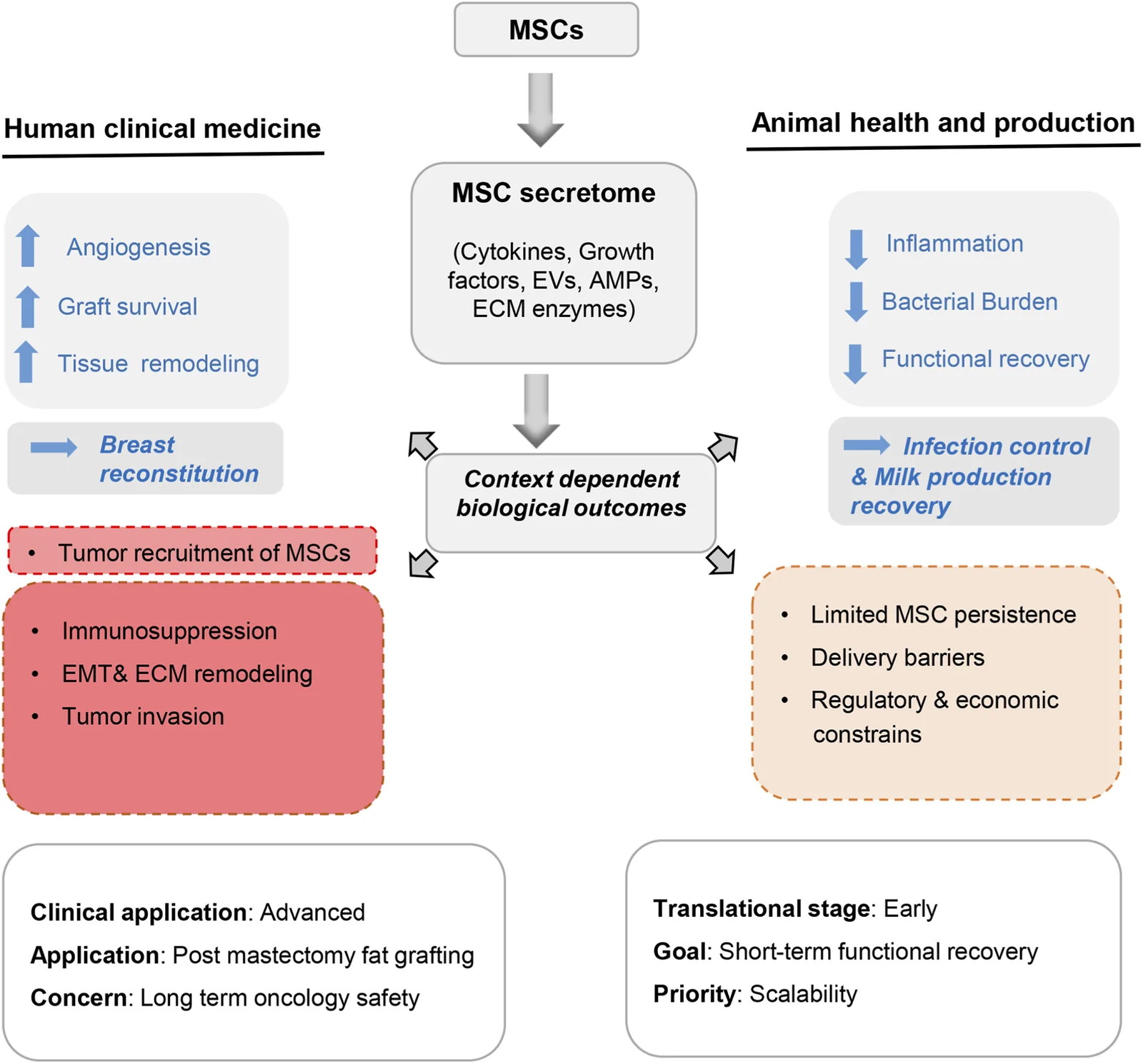
Context-dependent effects of mammary mesenchymal stem cells in human medicine and large farm animal health. Schematic overview of the diverse biological effects of MSCs across different clinical and veterinary contexts. At the center of the scheme, MSCs exert their effects primarily through their secretome, including cytokines, growth factors, EVs, antimicrobial peptides, extracellular matrix components, and regulatory enzymes. Their biological effects are highly context-dependent and shaped by the local microenvironment. In human medicine (left panel), MSCs have been extensively investigated for their regenerative and immunomodulatory properties. Reported effects include enhanced angiogenesis, improved graft survival, and tissue remodeling during regenerative processes. A prominent translational application is breast reconstruction following mastectomy, where MSC-enriched adipose grafts may enhance vascularization and tissue regeneration. However, the same stromal-supportive properties may have adverse consequences in oncological settings. MSCs can be recruited to tumor sites, where they may promote immunosuppression, extracellular matrix remodeling, epithelial-to-mesenchymal transition, and tumor invasion and progression. Thus, despite the therapeutic potential of MSC-based approaches for tissue regeneration, long-term oncological safety remains an important consideration, particularly in patients with a history of breast cancer. In bovine mammary health applications (right panel), MSCs are being explored experimentally as potential therapeutic tools to enhance tissue repair and restore mammary health. Reported effects include anti-inflammatory activity, reduced bacterial burden, and improved recovery following mastitis, highlighting their immunomodulatory and regenerative potential. However, translation into routine agricultural practice remains limited by challenges including *in vivo* persistence, delivery strategies, regulatory requirements, and economic feasibility. The translational stage of these applications differs across contexts and should not be interpreted as equivalent to established clinical or veterinary practice. In this schematic, "MSCs” is used as a broad functional term encompassing classical MSCs and related stromal cell populations described in clinical, veterinary, and experimental studies. AMPs- Antimicrobial peptides. EVs–Extracellular vesicles.

MSC–epithelial crosstalk can also affect tumor progression through pathways that are not necessarily dependent on EMT. In breast cancer, tumor cells recruit MSCs into the tumor microenvironment, where they can enhance metastatic potential through chemokine signaling, particularly via CCL5-dependent pathways (Karnoub et al., 2007). MSCs may also acquire carcinoma-associated fibroblast (CAF)-like phenotypes that contribute to a tumor-supportive niche (Mishra et al., 2008; Spaeth et al., 2009). These tumor-associated properties are relevant to the therapeutic use of adipose-derived tissues and cells in humans, including breast reconstruction (Figure 4). In breast reconstruction after mastectomy, autologous fat grafting has emerged as an alternative to prosthetic implants (Weaver et al., 2002; Thottapillil et al., 2025; Plaks et al., 2015). Importantly, several distinct adipose-based approaches should be differentiated. Conventional autologous fat grafting involves transplantation of harvested adipose tissue containing its endogenous stromal vascular cells, without deliberate isolation or enrichment of these cells. In contrast, SVF-enriched fat grafting involves processing adipose tissue to isolate the stromal vascular fraction and deliberately enriching the graft with this heterogeneous cell population. Cell-assisted lipotransfer (CAL) refers to fat grafting in which additional adipose-derived cellular material is incorporated, and reported approaches have included freshly isolated SVF and adipose-derived stromal/stem cells (ADSCs) (Yo et al., 2008; Shukla et al., 2020). These approaches differ in cellular composition and degree of manipulation and should therefore not be considered equivalent to administration of isolated, culture-expanded ADSCs, in which adipose-derived cells are expanded *ex vivo* before administration. Reported benefits of cell-enriched approaches include improved graft retention, vascularization, and integration.

The distinction among these interventions is also important when considering oncological safety. Mechanistic and preclinical studies have identified potential interactions between stromal cells and tumor-associated processes, including EMT, matrix remodeling, angiogenesis, and immunomodulation. However, these findings should not be extrapolated directly to all forms of adipose-based reconstruction. The cellular composition, processing, and degree of *ex vivo* manipulation differ substantially among conventional fat grafting, SVF-enriched grafting/CAL, and administration of isolated culture-expanded ADSCs. Clinical outcome evidence should therefore be considered separately for each intervention when evaluating potential oncological risks.

The consequences of MSC–mediated EMT differ between species (Figure 4). In humans, where breast cancer incidence is relatively high, MSC recruitment and EMT-associated plasticity are closely linked to malignancy, progression, and therapeutic resistance. In contrast, in bovine mammary tissue—where malignant transformation is rare—EMT-like programs have been reported under inflammatory conditions such as mastitis, although their broader significance remains uncertain. Experimental studies demonstrate that inflammatory stimuli, including LPS and stromal-derived signals, induce EMT-like changes in bovine mammary epithelial cells via pathways such as TGF-β1/Smad and NF-κB signaling, with associated extracellular-matrix deposition and fibrotic remodeling (Chen et al., 2017). These findings associate EMT-like plasticity with extracellular-matrix deposition, fibrotic remodeling, and impaired epithelial function in the experimental context examined, but do not establish a general species-wide relationship between EMT and fibrosis or exclude other consequences of EMT-like responses. Collectively, exogenous MSCs can modulate epithelial behavior through paracrine, extracellular-vesicle-mediated, and matrix-dependent mechanisms. Their capacity to modify tissue repair, epithelial plasticity, angiogenesis, and immune responses underlies their therapeutic potential. In the human breast, however, these same properties may also affect tumor-associated processes, providing a mechanistic rationale for continued evaluation of oncological safety. The relevance of these mechanisms should be considered in relation to the specific cellular intervention and its degree of manipulation. Available clinical evidence should therefore be considered alongside mechanistic and preclinical findings when evaluating the safety of MSC-based or adipose-derived cellular approaches.

### 5.4 MSC-based approaches for bovine mastitis: mechanistic basis and therapeutic potential

MSCs are being investigated experimentally as a potential therapeutic approach for mastitis in farm animals. Mastitis is a prevalent and economically significant inflammatory disease of the mammary gland. The disease is commonly detected through elevated somatic cell counts (SCC), and is frequently associated with reduced milk yield, impaired fertility, decreased body condition, and increased risk of premature culling (Stanek et al., 2024). Milk yield decreases by approximately 1–2 kg/day for each logarithmic increase in SCC once it exceeds 400,000–500,000 cells/mL. In primiparous cows, an SCC of 500,000 cells/mL is associated with a reduction in milk yield of 0.7–2.0 kg/day (3%–9%), depending on lactation stage (Koldeweij et al., 1999; Hagnestam-Nielsen et al., 2009). Although antibiotics remain the standard treatment for chronic infections, particularly those caused by *Staphylococcus aureus*, can be refractory, prompting investigation of additional therapeutic strategies, including immunomodulatory approaches such as the use of pegylated granulocyte-colony stimulating factor (PEG-G-CSF) (Putz et al., 2020; Timonen et al., 2021; Powell et al., 2018). MSCs are also being investigated experimentally as a potential therapeutic approach for mastitis. In Holstein–Friesian cows, intramammary or combined intramammary and systemic administration of allogeneic MSCs derived from bone marrow or adipose tissue reduced *S. aureus* counts and decreased milk SCC (Pokorska et al., 2024). Similarly, in murine models of bovine mastitis, systemic MSC infusion attenuated inflammatory responses, as reflected by reduced levels of TNFα, CXCL2, and ICAM-1 and increased IL-10 (Schouten et al., 2023). Notably, MSC engraftment in the mammary gland was not detected, likely due to pulmonary sequestration and systemic clearance. This suggests that therapeutic effects are mediated primarily through paracrine signaling rather than direct tissue integration (Fischer et al., 2009; Caplan et al., 2019). Consistent with this mechanism, MSCs promote immune regulation through the secretion of anti-inflammatory mediators, including IL-10, TGF-β, and prostaglandin E2 (Zhou and Bai, 2022). They also enhance innate immune function by modulating neutrophil and macrophage activity, thereby promoting pathogen clearance while limiting excessive production of TNF-α, IL-1β, and IL-6 (Ren et al., 2008; Kim et al., 2014). In parallel, MSCs support the expansion of regulatory immune populations, including CD4^+^ regulatory T cells and M2-polarized macrophages, further promoting the resolution of inflammation (Luque-Campos et al., 2021; Mittal et al., 2022). These paracrine mechanisms may underlie the effects observed experimentally despite limited MSC retention in mammary (Figure 4).

MSCs also exert direct antimicrobial activity through secreted antimicrobial peptides and proteins (Harman et al., 2022). These antimicrobial and immunomodulatory activities are further supported by extracellular vesicles, including EVs**,** which deliver bioactive cargo such as anti-inflammatory microRNAs (e.g., miR-146a and miR-21) (Phinney and Pittenger, 2017; Hu et al., 2022). Beyond immune regulation and antimicrobial activity, MSCs may also contribute to tissue remodeling by promoting fibroblast production of type I and III collagen and elastin while suppressing myofibroblast differentiation (Samad et al., 2014; Qin et al., 2023; Ozsvar et al., 2015). This dual effect may be particularly relevant in mastitis, where limiting fibrosis is critical for preserving mammary architecture and maintaining long-term milk productivity (Figure 4).

From a translational perspective, the route of MSCs administration remains a major challenge. Systemic delivery is limited by pulmonary entrapment, whereas intramammary administration is constrained by the anatomical organization of the bovine udder, particularly the large cisternal compartment that may restrict uniform cell distribution within ductal and parenchymal structures. Potential strategies include hydrogel-based carriers or cell-free MSC-secretome approaches that bypass anatomical barriers. However, these approaches remain experimental, and their efficacy, safety, optimal cell source and dose, processing conditions, and delivery methods require further validation. For cell-free approaches, comparisons among experimental studies are further complicated by differences in MSC source and culture conditions, secretome or EV processing and isolation methods, EV characterization, dose normalization, and the use of appropriate cell-free or vehicle controls. A detailed methodological analysis of EV-based therapeutics is beyond the scope of this review. Collectively, across both human and agricultural contexts, the therapeutic impact of MSCs is likely to depend less on classical notions of stemness and engraftment and more on the precise, context-dependent harnessing of their paracrine and immunomodulatory functions.

## 6 Discussion

The evidence considered in this review supports a view of mammary stem-cell biology in which regenerative capacity is not a property of isolated cell populations, but emerges from epithelial hierarchies operating within a specialized and dynamic tissue environment. EpSCs contribute to epithelial renewal and lineage production, while MSCs and other stromal populations shape the signals, extracellular matrix, metabolic conditions, and inflammatory context that regulate these processes. Mammary stem-cell function should therefore be considered in relation to its tissue environment rather than as an intrinsic property of a single cell population.

Murine lineage-tracing studies have challenged the concept of a single, permanently defined bipotent stem-cell population, whereas transplantation and other functional assays have provided evidence that some mammary epithelial cells can generate both luminal and basal lineages (Van Keymeulen et al., 2011; Rios et al., 2014; Lin et al., 2025). However, each experimental approach addresses a different aspect of stem-cell biology and has important methodological limitations. Marker-based approaches identify phenotypically enriched populations but do not by themselves establish stemness, long-term self-renewal, or physiological lineage contribution. Transplantation assays demonstrate regenerative potential under experimentally imposed conditions, but the recipient environment, tissue injury, and selection imposed during transplantation may not reproduce normal mammary physiology. Lineage tracing provides more direct evidence of cellular contribution within the intact gland, but its interpretation depends on labeling specificity, efficiency, timing, and the physiological context of the traced cells. Organoid assays provide information on self-organization, proliferation, and differentiation potential *ex vivo*, while removing much of the native epithelial–stromal and immune environment. Similarly, single-cell and spatial analyses, including studies of milk-derived cells, reveal cellular heterogeneity and molecular states but do not by themselves establish lineage relationships, long-term self-renewal, or functional contribution *in vivo* (Nguyen et al., 2018; Colacino et al., 2018; Regan and Smalley, 2020; Kumar et al., 2023; Rosenbluth et al., 2020; Gray et al., 2023). In humans, these approaches therefore provide complementary but indirect evidence because long-term lineage contribution cannot currently be resolved in the intact breast. Similarly, bovine studies provide evidence for regenerative and progenitor-associated properties, but lack direct *in vivo* lineage resolution (Rauner and Barash, 2012; Rauner and Barash, 2016). Thus, stem-cell identity should be considered through complementary dimensions—including phenotype, regenerative capacity, lineage output, localization, and physiological responsiveness—rather than inferred from marker expression alone.

The bovine mammary gland provides a particularly informative physiological context because prolonged lactation imposes sustained secretory demand that differs substantially from the reproductive cycles of laboratory mice. However, limited resolution of bovine lineage relationships remains an important constraint. Changes in putative EpSC populations during development or lactation therefore cannot automatically be interpreted as changes in functional capacity. Linking cellular measurements to tissue-level outcomes and physiological constraints is essential if stem-cell biology is to inform our understanding of mammary function and production.

The mTOR findings reviewed here (Kosenko et al., 2022b; Kfir and Barash, 2019) illustrate this distinction. Metabolic regulation can modulate stem-cell activity, but its effects depend on pathway activity, timing, tissue architecture, and the surrounding niche. The different consequences of sustained versus intermittent mTOR inhibition indicate that pathway manipulation cannot be interpreted simply as “more” or “less” signaling. Importantly, increased stem-cell activity does not imply unlimited epithelial expansion or proportional increases in milk production. Tissue architecture, metabolic supply, biosynthetic capacity, and other physiological constraints ultimately determine the extent to which changes at the cellular level can be translated into meaningful effects on gland function. For dairy production, this distinction is particularly important: increases in stem/progenitor activity or milk-protein gene expression may enhance milk production, but their effects are likely to be constrained by the overall capacity of the mammary gland to support increased synthesis and secretion.

The comparison with calorie restriction further indicates that mammary stem-cell regulation cannot be attributed to a single intracellular pathway. Metabolic information is integrated across epithelial, stromal, and immune compartments, including macrophage- and stromal-derived signals (Kosenko et al., 2022b; Kfir and Barash, 2019; Kosenko et al., 2022c). This broader regulation may be particularly relevant to production because sustained lactation requires coordination between epithelial renewal, differentiation, nutrient utilization, tissue remodeling, and inflammatory control. Thus, interventions aimed at improving productive function will need to be evaluated not only for their effects on stem-cell populations but also for their consequences for tissue homeostasis and the physiological state of the gland.

Mammary health represents a complementary translational dimension. The same epithelial–stromal interactions that support tissue maintenance and regeneration can become maladaptive during inflammation, injury, and tumor-associated remodeling. EMT and related changes are therefore better viewed as context-dependent states of epithelial plasticity affected by stromal signaling and tissue remodeling than as a simple epithelial-to-mesenchymal conversion. Understanding these interactions may help identify mechanisms linking impaired regeneration, chronic inflammation, and disease, although the molecular relationships between these processes remain incompletely resolved.

Mammary MSCs may also have translational relevance, particularly in inflammatory and tissue-injury settings. Their contribution may depend more on paracrine, immunomodulatory, extracellular-matrix, and vascular effects than on durable replacement of damaged tissue. Importantly, resident mammary mesenchymal populations should be distinguished from ex vivo-expanded MSCs that originate from bone marrow, fat pad, or umbilical cord, as their origin, phenotype, and *in vivo* behavior may differ. Findings from administered MSCs therefore cannot automatically be used to define the physiological role of endogenous mammary stromal populations. Similarly, persistence or localization of administered MSCs does not by itself establish durable tissue replacement. For mammary health applications, therapeutic benefit should consequently be assessed in terms of tissue repair, inflammatory regulation, and restoration of gland function rather than simply cell persistence or engraftment.

From a translational perspective, the key challenge is to establish how stem-cell activity contributes to measurable outcomes in mammary gland function, health, and production. In dairy animals, these include sustained epithelial regeneration, appropriate responses to the demands of lactation, mammary integrity, resistance or recovery from inflammatory injury, and ultimately productive performance. Importantly, these outcomes are unlikely to be determined by stem-cell abundance alone. The timing and magnitude of epithelial renewal, its coordination with differentiation and secretory activity, and its interaction with stromal and immune compartments may be equally important.

Taken together, the comparative evidence argues against viewing mammary stem-cell populations, particularly EpSCs, simply as targets for expansion. A more useful objective is to understand how epithelial stem/progenitor cells and mesenchymal populations interact and contribute to maintaining a functional mammary gland across development, lactation, involution, and injury. Mouse models provide the greatest lineage resolution, human studies provide essential information on epithelial diversity and disease relevance, and bovine studies offer a physiologically distinctive large-animal context. The translational value of these models lies not in treating them as interchangeable, but in identifying which biological principles can inform mammary health and production across species.

Future progress will depend on integrating lineage- or functionally resolved measurements with single-cell and spatial characterization of epithelial and stromal stem cell populations, together with metabolic and inflammatory analyses. In the bovine gland, such integration could help distinguish changes that merely alter stem/progenitor-associated markers from those that produce durable changes in mammary function, health, and productive capacity. This distinction will be essential for translating advances in mammary stem-cell biology into biologically meaningful and practically relevant applications.
